# (In)Fertility in the Down syndrome

**DOI:** 10.1590/1806-9282.20240537

**Published:** 2024-08-30

**Authors:** Alessandra Bernadete Trovó de Marqui, Maria de Fátima Borges

**Affiliations:** 1 Universidade Federal do Triângulo Mineiro, Instituto de Ciências Biológicas e Naturais – Uberaba (MG), Brazil.; 2 Universidade Federal do Triângulo Mineiro, Instituto de Ciências da Saúde – Uberaba (MG), Brazil.

Down syndrome (DS) is the most frequent genetic cause of intellectual disability, and it occurs in approximately 1 of 800 births worldwide. The causes of DS include trisomy (95%), translocation (3–4%), mosaicism (1–2%), and partial trisomy (<1%)^1^. A recent review described genes that may play causal roles in DS phenotypes such as gene expression changes affecting brain development, cardiac tissues, and ocular development, which lead to myeloproliferative disease and endocrine diseases^2^.

A Brazilian study reported that DS was associated with maternal age ≥ 35 years, paternal age ≥ 30 years, the performance of six or more prenatal consultations, prematurity, and low birth weight (p<0.05)^3^. It is consensus in the scientific literature that the main established risk factor associated with DS is still advanced maternal age (≥ 35 years)^4^.

The average life expectancy for people with DS has substantially increased from 25 years in 1983 to 60 years in 2020 due to improvements in medical care. This way a clinical guideline with recommendations to support high-quality primary care for adults with DS is essential^5^. A systematic review highlighted the need to improve the quality of life in adults with DS. Most of them wanted to become more independent, have relationships, participate in the community, and exercise their human rights^6^.

Furthermore, because individuals with DS are living longer, the question of (in)fertility must also be considered.

The literature data suggest that fertility is impaired in people with DS. This genetic condition appears to cause spermatogenesis defects in men and premature menopause in women. Nevertheless, some people with DS have become parents, and others are seeking to have children. In these cases, one must consider the person’s ability to care for and educate a child and the rationale for access to assisted reproductive technology (ART) and/or oocyte donation programs. DS is associated with early dementia (early-onset Alzheimer’s disease, i.e., at around the age of 40 years) which significantly impairs personal independence and parenting abilities^7^.

In addition, the risk of transmission of DS differs dramatically for men vs. women with DS. According to the literature data, all the children fathered by a man with DS (with or without ART) have been healthy. In contrast, one out of three children born to a woman with DS themselves have DS. In summary, the intellectual disability, early dementia, and the transmission of the pathology to their offspring have a negative influence on parenting in the DS^7^.

Often men with DS are infertile. Until 2019, only three cases of spontaneous conception in men with DS have been described^7^. One of them is a case report published in 2006 that described a 26-year-old man with DS fathered a normal son and the paternity was proven by microsatellite marker analysis^8^. Another case report more recently reported that a 36-year-old man with 47,XY,+21 karyotype is the biological father of his two normal boys, and paternity analysis using 26 microsatellite loci confirmed this result^9^. These studies support the necessity of advising people responsible for the care of adults with DS about possible fertility.

Alnoman et al.^10^ utilized a population database to address the paucity of data around pregnancy outcomes in women with DS. Patients with DS were at increased risk of giving birth prematurely (aOR 3.09, 95%CI 2.06–4.62) and having adverse neonatal outcomes such as small for gestational age (aOR 2.70, 95%CI 1.54–4.73), intrauterine fetal demise (aOR 22.45, 95%CI 12.02–41.93), congenital anomalies (aOR 7.92, 95%CI 4.11–15.24), and fetal chromosomal abnormalities. Women with DS should be counseled preconceptionally about these risks, and increased antenatal surveillance is advised^10^.

The data presented here reinforce the importance of providing guidance to health professionals on fertility in DS, despite the fact that evidence of these individuals having descendants is rare. There is a paucity of data in the literature about (in)fertility in the DS, highlighting the need for more studies in this area.
